# Lamina Influences on Tensile Strength of Shallow Marine Shales from Upper Ordovician, Western Ordos Basin

**DOI:** 10.1021/acsomega.3c01954

**Published:** 2023-08-08

**Authors:** Duo Wang, Zhidi Liu

**Affiliations:** † College of Petroleum Engineering, Xi’an shiyou University, Xi’an, Shanxi 710065, China; ‡ College of Earth Science and Engineering, Xi’an shiyou University, Xi’an, Shanxi 710065, China

## Abstract

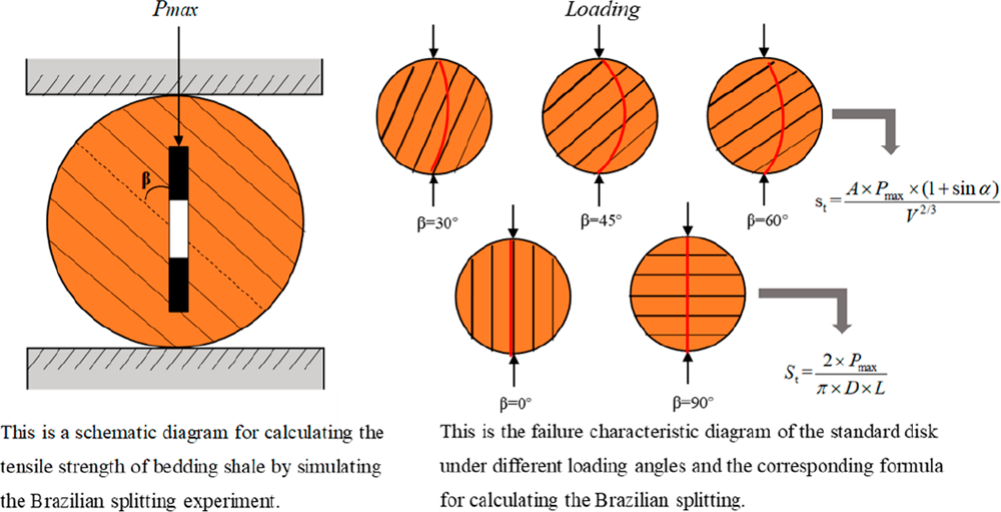

To investigate the influence of the lamina effect on the tensile strength of shallow marine shales and improve the shortcomings of the existing Brazilian standard disc splitting method, the Lamina shale of the Upper Ordovician Wulalik Fm in the western margin of the Ordos Basin was selected as the experimental object. Based on the radial wave velocity anisotropy test, the direction of crustal stress was determined, and standard cores were drilled. The Brazilian standard disc splitting experiment on Lamina shale with different loading angles was designed and carried out. The influence of lamina on the tensile strength of shale was summarized, and an improved calculation method of tensile strength was proposed. The experimental results indicate that the presence of lamina makes the tensile strength of shallow marine shale exhibit significant anisotropy, and the fracture surface morphology of standard discs under different loading angles varies greatly. The overall failure characteristics can be classified into two types: linear and curved. When the loading angle is 0° or 90°, the fracture surface of the disc belongs to tensile failure (linear type), and the traditional splitting method has good applicability. When the loading angle is greater than 0° and less than 90°, the fracture surface of the disc belongs to tensile shear failure (curve type), and traditional splitting methods are not applicable. There is a difference in tensile strength between vertical and horizontal wells, and vertical wells should consider the comprehensive tensile strength of the rock matrix and lamina at a 90° loading angle. Horizontal wells should consider the tensile strength of the weak lamina plane with a loading angle of 0°. The improved Brazilian splitting method solves the problem of the traditional method, calculating lower tensile strength values when the loading angle is greater than 0° and less than 90°. This provides important basic data support for wellbore stability evaluation and reservoir stimulation transformation.

## 1 Introduction

Shallow marine shale gas exploration in the Ordos Basin in northwest China continues to deepen, and the development of shale lamina in the Wulalik Fm of the Upper Ordovician in the west of the basin leads to prominent anisotropy (Gallant et al., 2007; Chen et al., 2019).^1,2^ This study aims to use exploratory experimental methods to describe the failure characteristics and tensile strength data changes of layered shale in order to discover the shortcomings of traditional Brazilian fracturing methods and improve them in order to provide reliable tensile strength data support for large-scale volume fracturing in engineering practices.

International and domestic scholars have done a great deal of fruitful work on the anisotropic characteristics of the tensile strength of layered rock masses. Since the end of the 1950s, the mechanical research of anisotropic rocks has attracted people’s attention. Donath (1961), Chenevert et al. (1965), and Mclamore et al. (1967) have devoted most of their early work to the anisotropic research of the compression behavior of layered rocks,^3−5^ and Hobbs (1964), Barron (1971), Barla et al. (1973), and Chen (2001) focused on the Brazilian fracturing experiment of shale;^2,6−8^ therefore, studying the lamina effect of shale under different loading angles is helpful to reveal the actual tensile strength and failure characteristics of shale, so as to better serve engineering practices. Lisjak et al. (2014) studied the anisotropy of the tensile strength of shale, schist, and gneiss by using the Brazilian splitting experiment and analyzed the influence of Lamina on shale failure mode;^9^ Chen et al. (1998) established the functional relationship between splitting tensile strength and rock parameters.^10^ Based on the research of Chen et al., Claesson (2002) proposed the analytical solution of splitting tensile strength and rock parameters as well as lamina angle. He believed that the direction of lamina (schistosity) has a significant impact on the tensile strength and failure mode of rock mass, which also reflects the anisotropic characteristics of the tensile strength of layered (schistose) rock.^11^ Ramamurthy et al. (1993) studied and analyzed the influence of anisotropy of layered rock mass on strength and modulus.^12^ Nasserimbh et al. (2003) analyzed the anisotropic mechanical properties of schist strength and deformation based on experiments.^13^ Cho et al. (2012) studied the anisotropy of elastic parameters and strength of three kinds of rocks through uniaxial compression and Brazilian splitting experiments of gneiss, shale, and schist at different angles.^14^ Tavallali et al. (2016) and Hu et al. (2021) through research found that the direction of bedding has a significant impact on the tensile strength and fracture characteristics of shale, and shales with different bedding directions have different fracture modes and fracture surface morphologies.^15,16^ Hoek (1964) carried out the pioneering work of direct tensile experimentation on slate and pointed out that the tensile strength parallel to the lamina plane is far greater than that perpendicular to the lamina plane;^17^ Youash et al. (1966) and Goffi (1974) carried out a series of direct tensile experiments on anisotropic rocks (including gneiss, shale, and two sandstones), which verified the significant influence of the Lamina plane on the tensile strength and failure mode, conducting a similar direct tensile study on layered schist and gneiss.^18,19^ Jensen (2016) and Jin et al. (2018) carried out a series of direct tensile experiments on shale samples with different Lamina angles; the influence of anisotropy on tensile strength and failure mode is proved. However, the current direct tensile shale database is limited, and the anisotropic failure mechanism is still unclear.^20,21^ Zuo et al. (2020) studied the mechanical anisotropy and failure modes of layered dolomite samples through UC experiments and classified five failure modes (rock tensile cracking, rock shear failure, lamina cracking, lamina sliding failure, and rock bending);^22^ Debecker et al. (2013) carried out experimental and numerical research on the failure mode of layered slate and analyzed the influence of the strength anisotropy of slate.^23^ Tan et al. (2015) considered the influence of Lamina angle on shale bearing capacity and fracturing behavior and proposed the common mixed failure mode in heterogeneous shale.^24^ Yang et al. (2015) studied the anisotropy of shale strength and failure mode based on Brazilian fracturing experiment.^25^ Jia et al. (2013), Xu et al. (2013), and Chen et al. (2014) carried out mechanical experiments of shale Lamina surface coring from different angles and obtained its mechanical parameters and failure mode characteristics.^26−28^ Liu et al. (2012), Liu et al. (2013), and Tan et al. (2014), taking into account different Lamina angles, conducted Brazilian splitting experiments on slate, chlorite schist, shale, and gneiss (with significant Lamina distribution), respectively, to study the influence of Lamina direction on the tensile strength and failure mode of layered rock mass.^29−31^

Based on previous research results, Li et al.’s (2022) shale comprises a heterogeneous and typically laminated fine-grained assemblage of minerals imparting both strong anisotropy and low permeability. Such strong heterogeneity and anisotropy in rock mechanics properties provide strong control over the shallow marine shales.^32,33^ It is necessary to conduct tensile strength experiments on shale under the influence of Lamina effects (this study uses Brazilian splitting experiments conducted on standard discs at different loading angles as a means of reflecting Lamina effects, where “loading angle” refers to the angle between the loading line and Lamina direction). As the most widely used method for obtaining rock tensile strength both domestically and internationally, many scholars have proposed various measures for the splitting method in recent years, including limiting the thickness to diameter ratio of the standard disc. However, these measures are aimed only at adjusting the rock sample preparation and experimental plan and do not involve the improvement of calculation formulas. At present, the Brazilian standard disc splitting experiment usually only considers the case where the loading angle is 0° (the loading line and lamina direction are consistent), and some even do not consider the impact of the loading angle on the tensile strength, which is only randomly measured. In engineering practice, rock occurrence is complex and changeable (such as horizontal structure, high and steep structure, etc.), and its stress forms are diverse. In particular, which direction of tensile strength should be used for shale gas vertical wells and horizontal wells is still worth discussing.

Therefore, it is urgent to clarify the influence of lamina on the tensile strength of shale, including the size and failure characteristics of lamina on the tensile strength. In addition, it is necessary to discover and improve the shortcomings of the existing Brazilian standard disc splitting method in order to accurately calculate the anisotropic tensile strength of shallow marine Lamina shale and provide important basic data support for wellbore stability evaluation and reservoir stimulation and transformation.

## 2 Geological Setting

**Figure 1:**
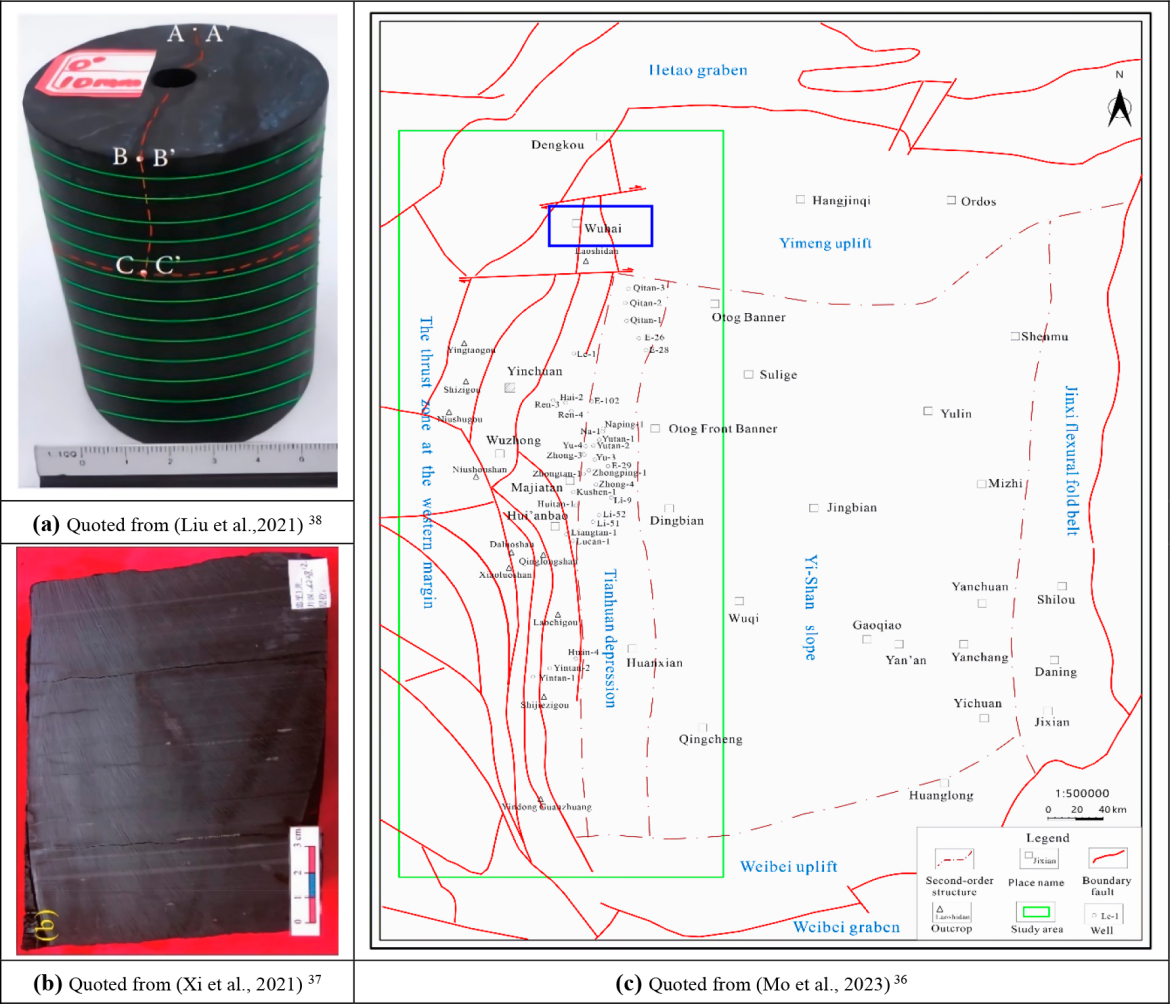
Geographical location map of the Zhuozi Mountain area in the Tianhuan Depression on the western edge of the Ordos Basin and the shale coring samples from the Wulalik Fm (part (a) courtesy of Yang Liu et al.,^38^ Copyright 2021; part (b) courtesy of Shengli Xi et al.,^37^ Copyright 2021; part (c) courtesy of Wuling Mo et al.,^36^ Copyright 2023): (a) and (b) are full-diameter shale coring samples and lamina cross sections perpendicular to the transverse isotropy; (c) is the geographical location of the Wulalik Fm in the Zhuozi Mountain area of the study area.

The Ordos Basin in northwest China geographically spans four provinces and regions, including Shanxi, Gansu, Ningxia, and Inner Mongolia. It is located at the junction of six tectonic units, including the North Shanxi Slope Zone, the Jinxi flexual fold belt, the Yimeng uplift, the Weibei uplift, the western margin thrust belt, and the Tianhuan depression.^34,35^ The research area is located in the Zhuozi Mountain area of the Tianhuan Depression in the western part of the Ordos Basin, northwest China. It is generally distributed in a north–south direction, with an east–west width of 50–200 km and a north–south length of about 600km, for an area of approximately 520 km^2^. The thickness and reserves of the shale layers in the Wulalik Fm are very considerable, with an average shale thickness of 50–100 m and a maximum of 400 m, with a total reserve of approximately 60 billion cubic meters;^36^ the geographical location of the study area is shown in Figure 1c.

The Wulalik Fm in the Ordos Basin is a set of sedimentary strata in the early Late Palaeozoic in northern China. Shallow marine shale is an important part of the Wulalik Fm (shallow marine shale refers to the shale deposited in the sea area with relatively shallow water depth, which is usually closely related to the sediment supply, seawater environment, water depth, and other factors). The sedimentary environment of this group is complex and diverse, including various sedimentary facies, such as nearshore shallow seas and bays. Among them, mudstone and shale in nearshore shallow sea environments are mainly composed of terrestrial materials, marine biological debris, and debris materials; Shale in the bay environment has a higher organic matter content and pyrolysis gas yield. The shale sedimentary layer is mainly composed of grayish black carbon, siliceous mudstone, carbonaceous mudstone, and carbonaceous mudstone containing lime mud. In the middle, there are several layers of gravel limestone, which are rich in graptolites and develop striped bands. The gravel, limestone, and mudstone show abrupt contact without a continuous lithological transition. It is a deep-water basin facies and contains carbonate gravity flow sedimentation.

Therefore, the shale of the Wulalik Fm is mainly composed of black shale and gray shale, which are rich in organic matter. It contains abundant biological fossils and organic matter, with an organic matter content of up to 2%–15%, and has good pyrolysis performance. In addition, Wulalik Fm shale is also characterized by large thickness, rich reserves, and good geological reservoir conditions and is one of the important target horizons for shale gas exploration and development in the Ordos Basin. This set of shale foliations is relatively developed. A large number of graptolite fossils can be seen on the core section, and the burial depth is 1500–2300 m.^37,38^ According to the previous exploration results, the high-quality shale of Wulalik Fm is relatively developed in the low-lying zone of the deep water slope in the middle north section of the western edge of the basin, with good physical properties, mainly composed of shale, quartz, feldspar, etc., of which the shale content is more than 80%, which is a favorable area for shale gas exploration. However, the degree of thermal evolution of the southern section is low, and it is only a prospective area for shale gas exploration. In the longitudinal direction, favorable source rocks are mainly distributed at 20–30 m from the bottom of the Wulalik Fm. The cross section and full diameter rock samples of Wulalik Fm shale samples are shown in Figures 1a,b.

In other words, Wulalik Fm has rich sedimentary materials and a unique sedimentary environment, forming excellent shale gas reservoirs that will provide important resource bases and technical support for the exploration and development of shale gas in the Ordos Basin.

## 3 Experimental Materials and Methods

The experimental study includes two parts. The first part is the radial wave velocity anisotropy test of laminated shale, which aims to determine the maximum horizontal principal stress direction of the full-diameter rock sample used and provide the direction basis for drilling the standard core. The second part is the tensile strength experiment of laminated shale, which aims to obtain the tensile strength data and failure characteristics of the standard disc at different loading angles.

### 3.1 Testing the Principle of Radial Wave Velocity Anisotropy in Laminated Shale

The crustal stress direction is the azimuth of the horizontal principal stress direction, which plays an important role in the development of oil and gas fields. The crustal stress direction can be determined by core analysis, logging data interpretation, seismic data interpretation, and other methods. Among them, core analysis has different measuring principles, such as wave velocity anisotropy, acoustic emission, and differential strain analysis. Using the wave velocity anisotropy method to determine the direction of core crustal stress is the most convenient and commonly used test method. The basic principle of determining the crustal stress by the anisotropy of rock wave velocity is that the rock in the formation is in a state of three-dimensional stress. When the core is separated from the original stress state during drilling and coring, the stress will be released by itself. In the process of stress release, the rock will form microfractures in proportion to the degree of unloading of the core. The degree of development of microfractures has an inherent genetic relationship with the size and direction of crustal stress.

The cracks formed by stress release are filled by air, and the wave resistance values of rock and air are very different; therefore, the existence of dominant small cracks in the rock core makes the propagation speed of sound waves in different directions of the rock core different; there is obvious anisotropy; and the propagation speed of sound waves in the direction of the maximum principal stress of rock is the slowest. On the contrary, in the direction of the minimum stress, the acoustic wave propagation speed is the fastest. Using the above principle, we can obtain the propagation velocity of sound waves in different directions can be obtained. The slowest direction is the direction of the maximum horizontal principal stress.

### 3.2 Test Device and Program Flow

#### 3.2.1 Test Device

**Figure 2:**
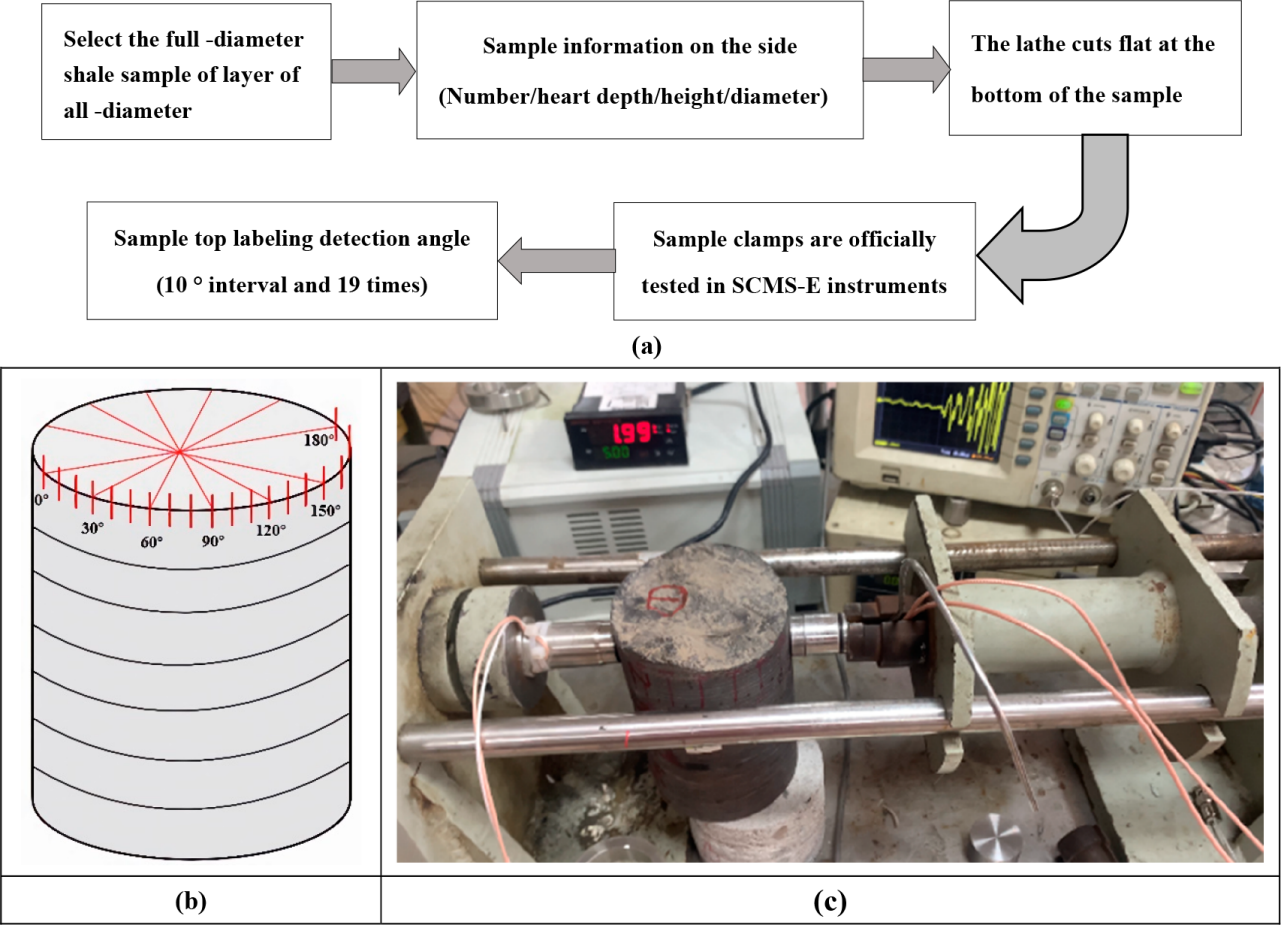
(1-39/69) Overall scheme for acoustic anisotropy testing of full-diameter rock samples: (a) specific flow of radial acoustic anisotropy testing of rock samples; (b) marks for the direction of rock sample testing; and (c) scanning rock samples for the SCMS-E high temperature and high pressure core multiparameter acoustic detector.

The wave velocity anisotropy testing device is the SCMS-E high temperature and high pressure core multiparameter acoustic detector, as shown in Figure 2c, which is mainly composed of a core holder, an ultrasonic probe, an ultrasonic generator, an oscilloscope, and other parts. During the test, the ultrasonic probe is coated with a coupling agent and closely connected to the core. A group of sound waves is generated by the ultrasonic generator and passes through the core through the transmitting end. The acoustic signal is obtained from the receiving end through the oscilloscope analysis, and then, the propagation rate of the ultrasonic wave in the core is calculated using the acoustic transmission time. The rotary core holder is marked with an angle scale, and the acoustic velocity of the core at different angles can be measured by rotation.

#### 3.2.2 Scheme Process

The full-diameter shale used in the experiment was taken from the Upper Ordovician Wulalik Fm in the western margin of the Ordos Basin, with obvious lamina development and belonging to argillaceous shale, with a core depth of 2796.06 m, No. 1-39/69, a height of 15 cm, and a diameter of 10.2 cm. In order to control the dispersion of the experiment results and eliminate the interference of unrelated factors with the experiment as much as possible, the SCMS-E type high temperature and high pressure core multiparameter acoustic detector is used to conduct the radial wave velocity anisotropy test of full-diameter rock samples. The test process and direction marking are shown in Figure 2a,b. The purpose of selecting a direction with stable physical properties to drill the standard core (height 50 mm, diameter 25 mm) is to determine the direction with the maximum velocity of the P and S waves (minimum horizontal principal stress direction) so as to eliminate the interference of shale in the process of sedimentary diagenesis due to the irregular arrangement of constituent minerals and the directional arrangement of fractures and microcracks. The specific steps of the test are as follows: 1: cut the bottom end of the full-diameter rock sample with a processing lathe and place it at the detection position of the bottom plate of the acoustic detector; 2: calibrate the detection angle at the other end of the rock sample 1.5 cm from the upper part, from 0° to 180°, and every 10° to find the maximum value of the longitudinal and transverse wave velocity in the circumferential direction.

### 3.3 Test Results of Wave Velocity Anisotropy

**Figure 3:**
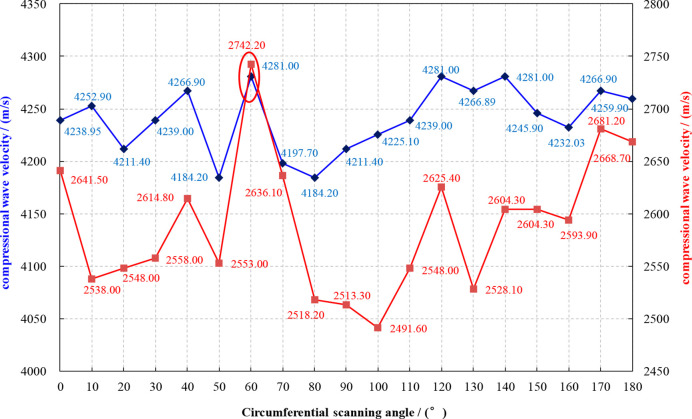
(1-39/69) Relationship between the P-wave velocity and the circumferential scanning angle of the full-diameter rock sample.

According to the test scheme, the test results of longitudinal and transverse wave velocities between 0° and 180° are obtained, as shown in Table 1 and Figure 3.

**Table 1:** Test Results of Wave Velocity Anisotropy

Core No.	Depth (m)	Altitude (cm)	Diameter (cm)	Angle (deg)	Longitudinal wave time (μs)	Shear wave time (μs)	Longitudinal wave velocity (m/s)	Shear wave velocity (m/s)
1-39/69	2796.06	15	10.20	0	24.44	39.22	4238.95	2641.50
1-39/69	2796.06	15	10.20	10	24.36	40.82	4252.90	2538.00
1-39/69	2796.06	15	10.20	20	24.60	40.66	4211.40	2548.00
1-39/69	2796.06	15	10.20	30	24.44	40.50	4239.00	2558.00
1-39/69	2796.06	15	10.20	40	24.28	39.62	4266.90	2614.80
1-39/69	2796.06	15	10.20	50	24.76	40.58	4184.20	2553.00
1-39/69	2796.06	15	10.20	60	24.20	37.78	4281.00	2742.20
1-39/69	2796.06	15	10.20	70	24.68	39.30	4197.70	2636.10
1-39/69	2796.06	15	10.20	80	24.76	41.14	4184.20	2518.20
1-39/69	2796.06	15	10.20	90	24.60	41.22	4211.40	2513.30
1-39/69	2796.06	15	10.20	100	24.52	41.58	4225.10	2491.60
1-39/69	2796.06	15	10.20	110	24.44	40.66	4239.00	2548.00
1-39/69	2796.06	15	10.20	120	24.20	39.46	4281.00	2625.40
1-39/69	2796.06	15	10.20	130	24.28	40.98	4266.89	2528.10
1-39/69	2796.06	15	10.20	140	24.20	39.78	4281.00	2604.30
1-39/69	2796.06	15	10.20	150	24.40	39.78	4245.90	2604.30
1-39/69	2796.06	15	10.20	160	24.48	39.94	4232.03	2593.90
1-39/69	2796.06	15	10.20	170	24.28	38.64	4266.90	2681.20
1-39/69	2796.06	15	10.20	180	24.32	38.82	4259.90	2668.70

As shown in Table 1 and Figure 3, after 180° P-wave velocity scanning of 1-39/69 full-diameter rock samples, it can be seen that the P-wave velocity is 4281.00 m/s and the S-wave velocity is 2742.20 m/s in the 60° marked direction (marked in red circle), which are higher than the sound velocity in other directions. Therefore, it can be judged that the 60° marked direction is the minimum horizontal principal stress direction of the full-diameter rock sample, and its physical properties are the most stable. It can eliminate the interference of cracks and microcracks on the anisotropic tensile strength experiment.

### 3.4 Experiment Scheme for Tensile Strength of Laminated Shale

According to the standards of the International Society of Rock Mechanics, dark black laminated shale is collected and processed from the shallow marine Wulalik Fm in the western Ordos Basin and prepared by coring, cutting, and grinding a 50 mm × 25 mm standard disc specimen. The parallelism of the upper and lower surfaces of each experiment piece shall be controlled within 0.5 mm, and the flatness of the surface shall be controlled within 0.1 mm. All experimental pieces were stored in a dry environment at room temperature. The Brazilian standard disc splitting experiment involves a platform load. To ensure that the loading direction line passes through the center of the disc, first the specimen was marked in the preapplication direction, then the upper loading point was marked. When placing the specimen, the vertical line was used for calibration. After the specimen was fixed, the lower flat platform was also fixed and applied to the upper platform at a loading speed of 0.1 mm/min. At this time, we recorded and observed the stress–strain curve of the experiment. When the measured stress drops suddenly, the experiment piece forms cracks and stops loading, and then, the stress value is read at the moment and recorded.

**Figure 4:**
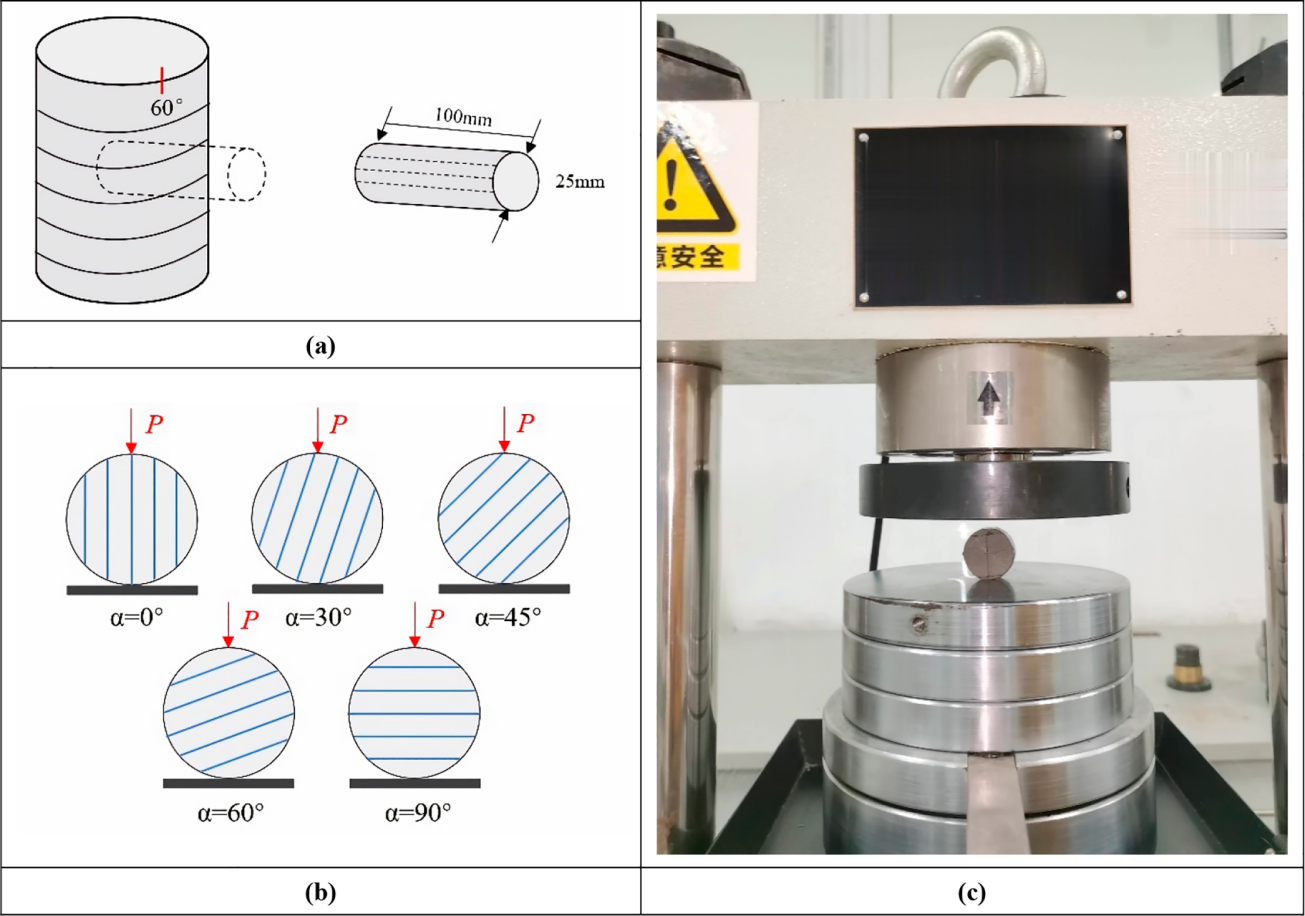
Preparation and experimentation of a shale standard disc under different loading angles: (a) schematic diagram of standard core sampling; (b) the standard disc under five different loading angles; (c) the standard disc split for the microcomputer-controlled constant stress pressure experiment machine.

For 1-39/69 full-diameter rock samples, a core (25 mm in diameter) was taken according to the 60° marked angle shown in Figure 4a, and the core direction is parallel to the lamina direction of the rock sample (horizontal sampling), which should be the core perpendicular to the transverse isotropic plane.

In order to minimize the influence of the size effect, it is necessary to strictly control the accuracy of the sample. If the height–diameter ratio is set to 1:2, the core is cut into a standard disc 12.5 mm high. In order to achieve uniform load distribution, the end parallelism error of all samples is limited to ±0.02 mm. In this experiment, the Brazilian splitting experiment is carried out on the rock sample perpendicular to the transverse isotropy in order to study the impact of loading angle on the tensile strength, deformation, and failure characteristics of shale and the angle between the loading line and the lamina plane. Five cases of α of 0°, 30°, 45°, 60°, and 90° are designed, and one standard disc is selected from each angle. Five standard discs are used to conduct Brazilian splitting experiments with a microcomputer-controlled constant stress pressure experimenting machine (type TYPC-OWC-300D) in the order of 0°, 30°, 45°, 60°, and 90°. The loading diagram and splitting experiment of the standard disc are shown in Figure 4b,c.

## 4 Results and Analysis

### 4.1 Experimental Results

This indoor experiment uses the traditional Brazilian standard disc splitting method to calculate the tensile strength of shale. It applies a concentrated load along the direction of the standard disc diameter, and the experiment piece will theoretically split along the direction of the axial force after being stressed (disc diameter). According to the theory of elastic mechanics, the horizontal tensile stress will be approximately uniformly distributed along the diameter direction of the concentrated force (*P*_c_). It is only necessary to replace *P*_c_ with the maximum compressive stress (*P*_max_) when the experiment piece is damaged, and *S*_t_ is calculated with formula 1:
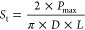
1where *S*_t_ is the tensile strength of the experiment piece, MPa; *P*_max_ is the maximum compressive stress when the experiment piece is damaged, MPa; *D* is the diameter of the experiment piece, mm; and *L* is the height of the experiment piece, mm.

**Figure 5:**
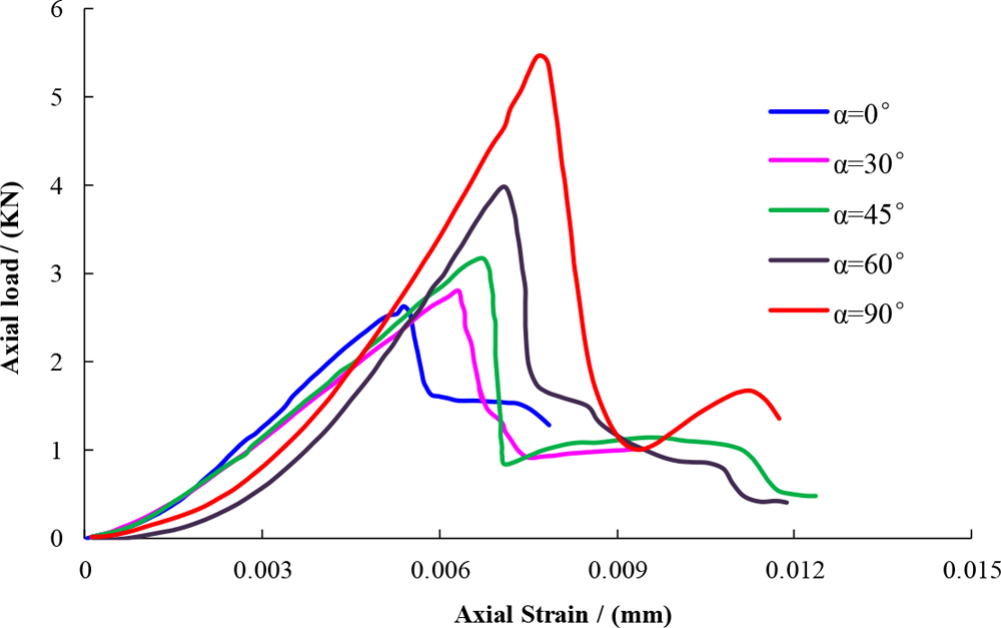
Stress–strain curves of a standard disk under different loading angles.

**Figure 6:**
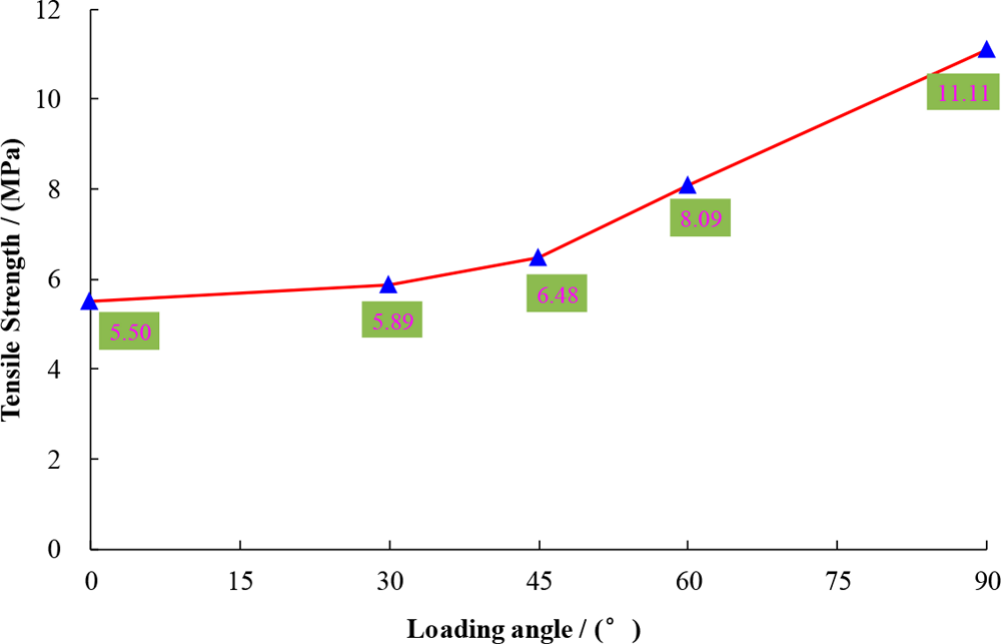
Change trend of the tensile strength of laminated shale under different loading angles.

According to the above experimental scheme and the traditional Brazilian standard disc splitting calculation formula, the tensile strength value and its change trend under different loading angles are obtained, as shown in Table 2, Figure 5, and Figure 6.

**Table 2:** Stress–Strain and Tensile Strength of Layered Shale under Different Loading Angles

Angle (deg)	*P*_max_ (kN)	Strain (mm)	*S*_t_ (MPa)
0°	2.70	0.005377	5.50
30°	2.89	0.006021	5.89
45°	3.18	0.006732	6.48
60°	3.97	0.007095	8.09
90°	5.45	0.007813	11.11

Figure 6 shows the stress–strain curves of the standard disc corresponding to five different loading angles. It is evident that as the loading angle increases both axial stress and strain increase, indicating that the lamina is very sensitive to axial load. The closer the lamina direction is applied with an axial load, the smaller the axial strain of the standard disc, which is prone to transverse tensile failure. This indicates that stress–strain is closely related to the tensile strength. Figure 5 shows that with the increase in stress and strain, the tensile strength of the standard disc also increases, indicating that the fundamental reason for the anisotropy of the shale tensile strength is due to the presence of lamina.

### 4.2 Influence of the Lamina Effect on the Tensile Strength of Shale

According to Table 2 and Figure 6, the influence of the loading angle on the tensile strength of shale can be analyzed. The tensile strength of shale increases with an increase in loading angle. If the loading angle is 0° (the loading line is parallel to the lamina), then the tensile strength is the minimum, 5.50 MPa. Because the lamina plane is a weak plane, the tensile strength is the minimum. The Brazilian split cracks appear along the lamina plane and pass through the center of the disc in a straight line. When the loading angle α is 90° (the loading line is perpendicular to the lamina), the maximum tensile strength is 11.11 MPa. Due to the joint action of the rock matrix and lamina and the fact that there is no weak plane or shear dislocation, Brazilian cleavage cracks appear along the vertical lamina plane and pass through the center of the disc in a straight line. It is calculated that the ratio of tensile strength in two vertical directions is 2.02, which shows that the impact of the loading angle on the tensile strength of shale is very significant. When the loading angle is 30°, 60°, or 90° (there is a certain angle between the loading line and the lamina), the tensile strength gradually increases; the rock matrix and lamina work together, and there are also weak planes and shear dislocations. The Brazilian splitting crack does not pass through the center of the disc in a straight line but in a curve shape, and its value is between 0° and 90° tensile strength.

### 4.3 Effect of Lamina on Shale Failure Characteristics

**Figure 7:**
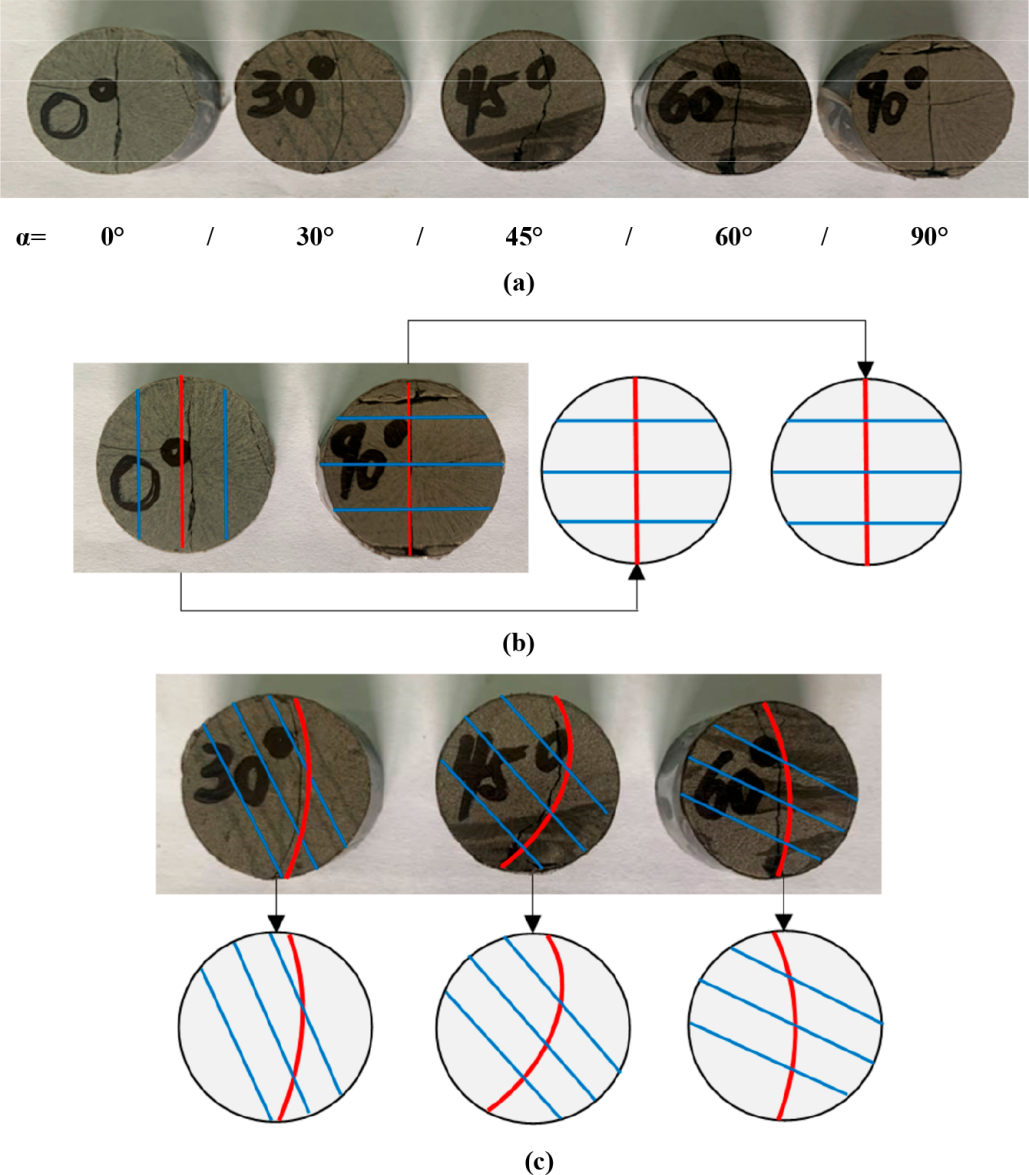
Failure characteristics of the standard disc under different loading angles: (a) the fracture morphology of five standard discs under different loading angles; (b, c) linear (α = 0° or 90°) or curved (α = 30°, 45°, 60°).

The fracture plane morphology of the standard discs of laminated shale after splitting at 0°, 30°, 45°, 60°, and 90° is shown in Figure 7. It can be seen from (a) that the fracture plane morphology of the five standard discs varies greatly under different loading angles. When it is 0° or 90°, the fracture plane is a regular plane passing through the loading line and the center of the disc. When it is 30°, 45°, or 60°, the fracture plane does not pass through the loading line and the center of the disc but presents a curved surface with a certain radius. Similar results were obtained from the Brazilian fracturing experiment on layered sandstone.^38^

As shown in Figure 7b,c, according to the development law of the fracture plane, two types of failure characteristics are summarized:

(1) When the loading angle is 0° or 90°, the fracture plane passes through the loading line and the center of the disc, forming a nearly regular plane. The Brazilian split crack is linear, which is a typical tensile failure. α = 0° corresponds to the tensile strength of the lamina plane, and α = 90° corresponds to the comprehensive tensile strength of the rock matrix and lamina plane.
(2) When the loading angle is greater than 0° but less than 90°, the fracture plane does not pass through the center of the disc but presents a curved surface with a radius. The Brazilian split crack is curved, which is a typical tension-shear failure. The reason is that the disc always tends to crack along the vertical direction near the loading line, but the straight section of the fracture plane is relatively short. Affected by the shear dislocation of the lamina, the fracture plane bends in the direction of the lamina and gradually turns into a curve. According to the loading angle range, it can be divided into two development characteristics:
  (a) When the loading angle increases from 0° to 45°, that is, greater than 0° but less than 45°, the straight section of the Brazilian cleavage crack gradually becomes shorter, the curve section gradually becomes longer, and the overall curvature of the crack increases. The bending degree of the crack reaches its maximum at 45°.
  (b) When the loading angle increases from 45° to 90°, that is, greater than 45° but less than 90°, the straight section of the Brazilian split crack gradually becomes longer, the curve section gradually becomes shorter, the overall bending of the crack decreases, and finally the fracture plane infinitely approaches the regular plane, that is, the fracture plane passes through the loading line and the center of the disc.

There are two kinds of typical failure characteristics in the Brazilian fracturing experiment of laminated shale: linear type and curve type. The basic reason for this is the distribution angle of the lamina plane and its relatively weak mechanical properties.

**Figure 8:**
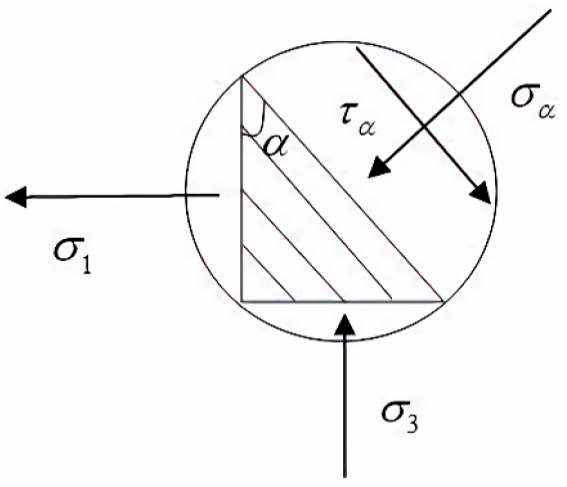
Schematic diagram of the lamina stress.

Brazilian splitting cracks always extend along the loading line and the center of the disc. When α is 0°, the loading line is collinear with its central lamina plane along the diameter of the disc. At this time, the central lamina plane of the disc has only tensile stress but no shear stress and the fracture plane extends in a straight line along the central lamina plane. At this time, the tensile strength of the lamina plane is obtained. When α is 90°, the loading line is perpendicular to the central lamina plane of the disc. At this time, the central lamina plane of the disc has only normal stress and no shear stress. The rock matrix and lamina plane jointly bear tensile stress. The fracture plane is expanded in a straight line along the loading line. At this time, the comprehensive tensile strength of the rock matrix and lamina plane is obtained. When the angle between the loading line and the lamina surface is greater than 0° but less than 90°, as shown in Figure 8, there is not only normal stress but also shear stress on the lamina plane.

According to the theory of elasticity, when the axial load is loaded with σ_3_, the experiment piece will produce horizontal tensile stress σ_1_. The Mohr–Coulomb strength criterion considers that, under the plane stress state, if there are two principal stresses acting on a certain point of rock samples σ_1_ and σ_3_, then normal and σ_1_ stresses exist. The expression is
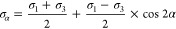
2
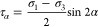
3where σ_α_ is normal stress, MPa; τ_α_ is shear stress, MPa; α Is the loading angle of rock sample, deg; σ_1_ is horizontal tensile stress, MPa; and σ_3_ is the axial load, MPa.

During the experiment, the axial load is the largest near the loading line and gradually decreases toward both ends along the direction perpendicular to the loading line, while the vertical axial load is the smallest near the center of the disc. Because the shear strength is relatively weak in the direction of the lamina plane, shear dislocation along the lamina plane is likely to occur near the center of the disc. From Formulas 2 and 3, when the angle is 45°, the shear stress on the lamina plane reaches the maximum, while the normal stress is the minimum, so the shear dislocation of the lamina plane is the most obvious, and the curve segment of the fracture plane reaches the longest at this time, with the maximum bending, which can better explain the law of the curve fracture plane changing with the loading angle in Figure 7c.

**Figure 9:**
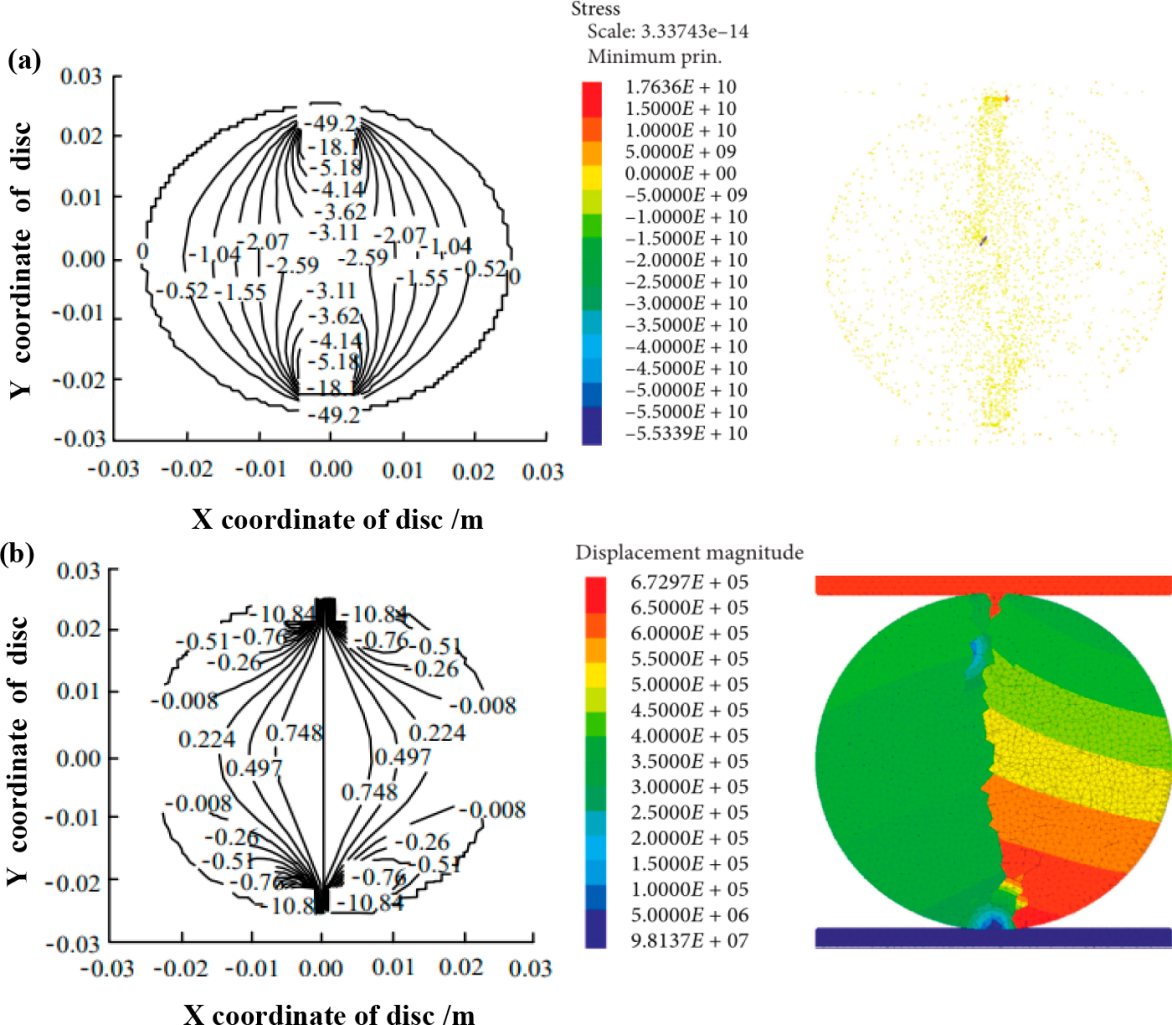
Simulation results of the disk model of non-laminated homogeneous shale under stress (left panels courtesy of Huafeng Deng et al.,^40^ Copyright 2016; right panels courtesy of Jiong Wang et al., Copyright 2021): (a) is the loading cloud of the dimensionless vertical stress and homogeneous model; (b) is the load–displacement cloud of dimensionless horizontal stress and the homogeneous model.

According to the description in the literature,^39−41^ the failure characteristics of the disc after splitting are explained by the stress field distribution during the experiment loading process, and the theory is used to verify the adequacy and correctness of the above analysis. As shown in Figure 9a,b, a homogeneous shale disc model without Lamina was created in 3DEC discrete element simulation software, which was used to load and record the nondimensional vertical stress field distribution characteristics of the disc and the loading nephogram of the homogeneous model, the nondimensional horizontal stress field distribution characteristics, and the load–displacement nephogram of the homogeneous model. It can be seen that the simulated splitting results are similar to the actual splitting results. This shows that both the vertical stress field and the horizontal stress field have important influences on the disc splitting shape. During the experimental loading process, the vertical tensile stress at the center point increases linearly with the loading displacement. Before the splitting failure, the stress increase trend was small. After cracking, the displacement field on the left side of the crack is larger than the displacement field on the right side. This is because the particles at the loading end are damaged after splitting, the uniformity of the disc is damaged, and the stress on both sides is uneven, resulting in an asymmetric distribution of the stress field.

## 5 Discussion

According to the above experiment results and analysis, the traditional Brazilian splitting method has a certain applicability in obtaining the anisotropic (Lamina) tensile strength of shale, but its limitations are also obvious, mainly because it is difficult to obtain accurate data within the range of a special loading angle. Therefore, this paper mainly introduces the specific scope of application of the traditional Brazilian splitting method and gives an improved calculation formula for its limitations.

### 5.1 Applicability Analysis

For the Brazilian standard disc splitting experiment, theoretically, the tensile stress in the horizontal direction of the disc is uniformly distributed in the plane on both sides of the loading line. The precondition for the calculation of formula 1 is also to assume that the starting point is at the center of the disc and that the fracture plane is a regular plane passing through the loading line and the center of the disc. Only if this condition is met will the corresponding actual mechanical deformation and tensile strength calculation formulas be consistent.

According to the previous failure feature analysis, the fracture plane of the disc has two types of tensile failure: linear type and tension-shear type. When the loading angle is greater than 0° but less than 90°, the fracture plane of the disc does not realize the central initiation, and the fracture position deviates from the diameter direction of the loading line. Therefore, the development law of the fracture plane of the disc cannot strictly meet the assumptions of the Brazilian standard disc splitting experimental mechanical model. The tensile strength value calculated by formula 1 is theoretically inappropriate and can only be an approximate value. Only when the loading angle is 0° or 90° is the tensile strength calculated by formula 1 theoretically accurate.

Therefore, there are applicable conditions for using the Brazilian standard disc splitting experiment to obtain the rock tensile strength. First, the lamina structure on both sides of the loading line of the disc sample must be symmetrically distributed, and the internal stress of the disc can meet the assumption of a symmetrical distribution under an axial load. The second requirement is that the failure characteristics comply with the tensile strength calculation formula, that is, at the loading angle is 0° or 90°, can be substituted into formula 1 for calculation; otherwise, the calculation result will have a large error, generally less than the true value. Therefore, the application scope of the traditional Brazilian splitting method cannot be simply and blindly expanded in the tensile strength experiments on laminated shale.

### 5.2 Method Improvement

When the loading angle is greater than 0° but less than 90°, the existence of axial load σ_3_ will produce mutually perpendicular normal stress on the Lamina plane σ_α_ (normal stress) and shear stress τ_α_. The resultant force formed by the two forces and the axial load are in the same direction. The real reason for the inaccurate calculation of the tensile strength is that the effect of the sum of the two forces on the experiment is not considered, but simply that the axial load in the direction of the loading line is the only stress that causes the disc splitting.

With desired normal stress σ_α_ and shear stress τ_α_, according to the Pythagorean theorem and the above eqs 2 and 3, the resultant force *F*_*T*_ can be expressed as
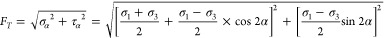
4

According to the requirements of the Brazilian disc splitting experiment, only axial load σ_3_ is applied externally during the experiment; here σ_1_ is due to σ_3_. The passive tensile stress in the horizontal direction is not the stress under active loading, so σ_1_ = 0, and then formula 4 is abbreviated as
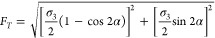
5

The calculation formula of the resultant force *F*_*T*_ can be obtained by further simplification:
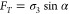
6

A disk with loading angle greater than 0° but less than 90° (curved fracture plane) can be defined as an irregular specimen due to its complex internal stress distribution. The volume needs to be introduced in the calculation, and the side area to participate in the calculation is only applicable to α = 0° or 90° (linear fracture plane). The resultant force *F*_*T*_ and axial load mentioned above, σ_3_, is on the same line and in the same direction, so the σ_3_ interaction with *F*_*T*_ is the root cause of the irregular fracture of the disk and σ_3_ is *P*_*max*_. Then the improved calculation formula of shale tensile strength applicable to the loading angle greater than 0° but less than 90° is
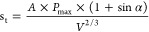
7where *A* is the stress correction factor (closely related to the height–-diameter ratio of the standard disc,; when the height–diameter ratio is 1:2, take 0.45); *V* is the volume of the experiment piece; α is the loading angle; and the value range is (0°, 90°).

The value of the stress correction coefficient A in eq 7 is based on the failure criterion of the unified strength theory, and the expression according to the failure criterion is
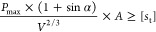
8

According to the failure criterion of the unified strength theory, the actual tensile stress must exceed the splitting strength *S*_t_ of the standard disk in order to cause failure. Therefore, the value of stress correction coefficient *A* is less than 1, and the value of *A* is determined by factors such as the size of the specimen. The expression for calculating *A* is as follows:
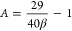
9where β is the aspect ratio, dimensionless.

**Figure 10:**
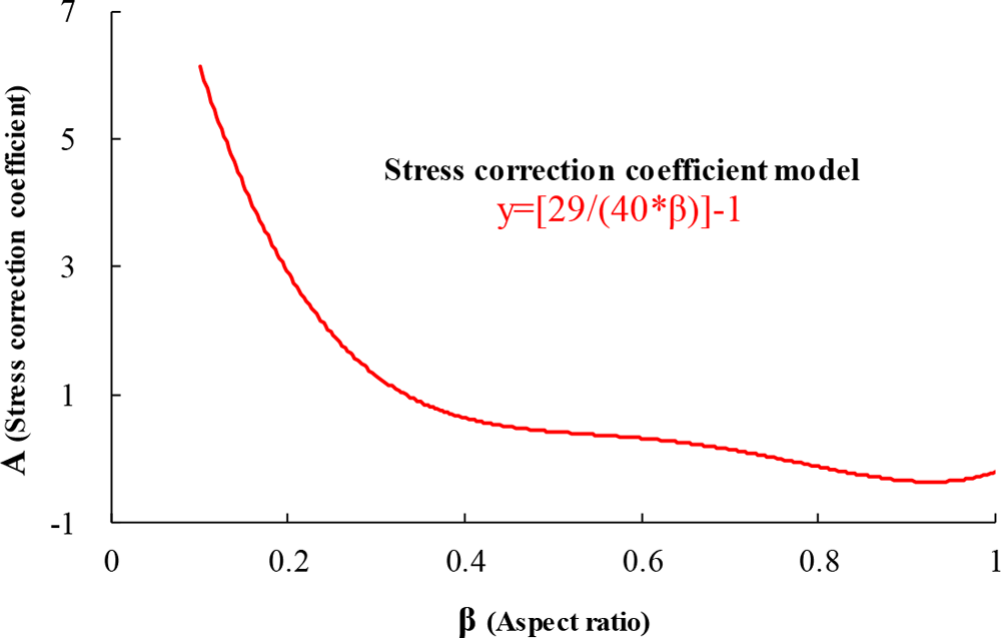
Relationship curve between the height to diameter ratio and stress correction coefficient.

Based on the constructed stress correction coefficient expression, a graph of the relationship between the aspect ratio and the stress correction coefficient has been formed (Figure 10), and it can be clearly read from the graph that when the aspect ratio β = 1:2, the stress correction coefficient *A* = 0.45. As the aspect ratio increases, the stress correction coefficient decreases. When the aspect ratio is 0.7, the stress correction coefficient begins to become negative; therefore, the setting of the standard disc aspect ratio should not exceed 0.7.

**Figure 11:**
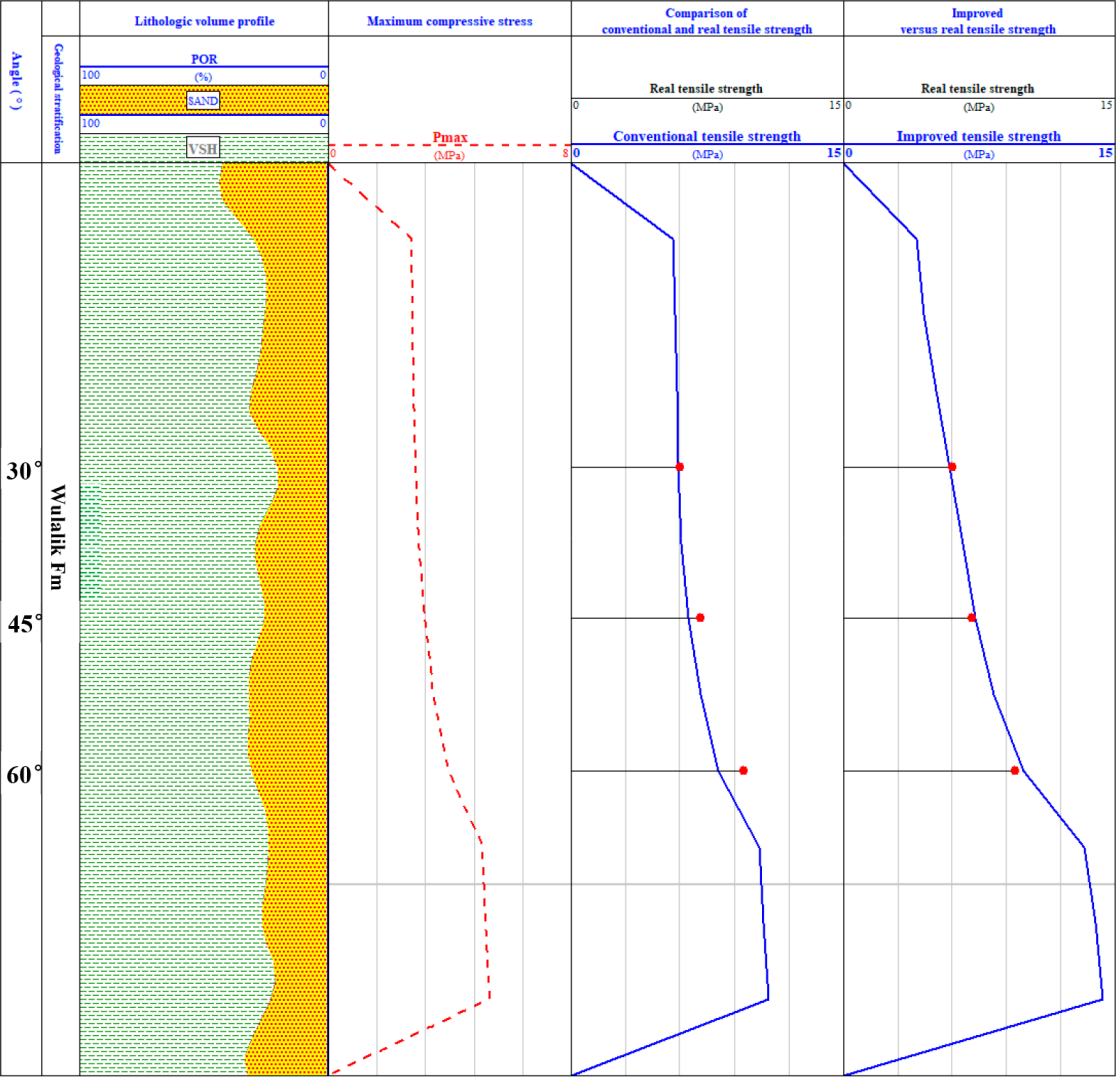
Comparative logging profiles of true tensile strength with traditional and improved methods at loading angles of 30°, 45°, and 60°.

With programming the above traditional and improved methods on the logging interpretation platform and calculating the corresponding tensile strength data according to this program, the accuracy and reliability of the improved method and the traditional method were verified by comparing them with the actual tensile strength measured by the direct method at loading angles of 30°, 60°, and 90°. The log interpretation comparison section is shown in Figure 11 (the red circle in the figure represents the actual tensile strength, the third blue curve represents the tensile strength data calculated by the traditional Brazilian splitting method, and the fourth blue curve represents the tensile strength data calculated by the improved Brazilian splitting method).

The actual tensile strengths corresponding to the loading angles of 30°, 60°, and 90° measured by the direct method are 6.08, 7.13, and 9.54 MPa, respectively. The relative errors of the tensile strength calculated by the traditional Brazilian splitting methods are −3.13%, −9.12%, and −15.20%, respectively, and the average relative error is −9.15%, which shows that the calculation result of the traditional Brazilian splitting method is seriously small. The relative errors of the tensile strength calculated by the improved Brazilian splitting method are 4.24%, 2.25%, and −4.29%, respectively, and the average relative error is 0.77%. It can be seen that the error in the calculation result of the improved Brazilian splitting method is small, and the result is very close to the true tensile strength value. The error analysis is shown in Table 3.

**Table 3:** Error Analysis of Tensile Strength Calculated by the Traditional Brazilian Splitting Method and Improved Method

Loading angle (deg)	*P*_max_ (kN)	Real tensile strength (MPa)	Conventional tensile strength (MPa)	Relative error	Improvement tensile strength (MPa)	Relative error
30°	2.89	6.08	5.89	–3.13%	5.82	–4.24%
45°	3.18	7.13	6.48	–9.12%	7.29	2.25%
60°	3.97	9.54	8.09	–15.20%	9.94	4.29%

Therefore, the new method corrects the tensile strength results accurately and solves the problem that the tensile strength results calculated by the traditional method are too small, which provides an important basic parameter for the later calculation of the formation pressure and even for the evaluation of the wellbore stability.

## 6 Conclusions

(1) The anisotropy of the tensile strength of laminated shale is very prominent. The impact of loading angle on the tensile strength of shale is basically the same, but the degree of impact is different. However, the fracture plane morphologies of disc samples with different loading angles are quite different, and the failure characteristics can be summarized into two types: linear type and curve type.
(2) When the loading angle is 0° or 90°, the fracture plane of the disc belongs to the tensile failure (linear type), which is a regular plane passing through the loading line and the center of the disc, meeting the assumptions of the mechanical model of the Brazilian disc splitting experiment. The tensile strength of shale calculated by the traditional splitting method is accurate. When the loading angle is greater than 0° but less than 90°, the fracture plane of the disc belongs to tension–shear failure (a curved line) and is not a regular plane passing through the loading line and the center of the disc. The corresponding actual mechanical deformation is not consistent with the traditional tensile strength calculation formula, so the application scope of the Brazilian splitting method cannot be simply and blindly expanded.
(3) The improved Brazilian splitting method takes into account the effect of the resultant force formed by normal stress and shear stress on the shale lamina plane on the axial load and also recognizes that the sample forming a curved fracture plane can be defined as an irregular sample, and it is more reasonable to introduce volume into the calculation. Its calculation formula solves the problem that the calculation value of the traditional formula is too small, which provides reliable basic parameters for later evaluation of wellbore stability.
(4) In engineering practice, the tensile strengths of vertical wells and horizontal wells are different. The comprehensive tensile strength of rock matrix and lamina corresponding to the loading angle of 90° should be considered for vertical wells, and its value is the maximum tensile strength. For horizontal wells, the tensile strength of lamina corresponding to the loading angle of 0° shall be considered, and its value is the minimum tensile strength.
